# Extending peripersonal space representation without tool-use: evidence from a combined behavioral-computational approach

**DOI:** 10.3389/fnbeh.2015.00004

**Published:** 2015-02-02

**Authors:** Andrea Serino, Elisa Canzoneri, Marilena Marzolla, Giuseppe di Pellegrino, Elisa Magosso

**Affiliations:** ^1^Laboratory of Cognitive Neuroscience, Department of Life Science, Center for Neuroprosthetics, Ecole Polytechnique Fédérale de LausanneLausanne, Switzerland; ^2^Dipartimento di Psicologia, Alma Mater Studiorum, Università di BolognaBologna, Italy; ^3^Dipartimento di Psicologia, Centro Studi e ricerche in Neuroscienze Cognitive, Polo Scientifico Didattico di Cesena, Alma Mater Studiorum, Università di BolognaBologna, Italy; ^4^Interdepartmental Centre for Industrial Research in Health Sciences and Technologies, Alma Mater Studiorum, University of BolognaBologna, Italy; ^5^Department of Electrical, Electronic and Information Engineering “Guglielmo Marconi,” Alma Mater Studiorum, University of BolognaBologna, Italy

**Keywords:** peripersonal space, tool-use, neural network model, multisensory processing, plasticity

## Abstract

Stimuli from different sensory modalities occurring on or close to the body are integrated in a multisensory representation of the space surrounding the body, i.e., peripersonal space (PPS). PPS dynamically modifies depending on experience, e.g., it extends after using a tool to reach far objects. However, the neural mechanism underlying PPS plasticity after tool use is largely unknown. Here we use a combined computational-behavioral approach to propose and test a possible mechanism accounting for PPS extension. We first present a neural network model simulating audio-tactile representation in the PPS around one hand. Simulation experiments showed that our model reproduced the main property of PPS neurons, i.e., selective multisensory response for stimuli occurring close to the hand. We used the neural network model to simulate the effects of a tool-use training. In terms of sensory inputs, tool use was conceptualized as a concurrent tactile stimulation from the hand, due to holding the tool, and an auditory stimulation from the far space, due to tool-mediated action. Results showed that after exposure to those inputs, PPS neurons responded also to multisensory stimuli far from the hand. The model thus suggests that synchronous pairing of tactile hand stimulation and auditory stimulation from the far space is sufficient to extend PPS, such as after tool-use. Such prediction was confirmed by a behavioral experiment, where we used an audio-tactile interaction paradigm to measure the boundaries of PPS representation. We found that PPS extended after synchronous tactile-hand stimulation and auditory-far stimulation in a group of healthy volunteers. Control experiments both in simulation and behavioral settings showed that the same amount of tactile and auditory inputs administered out of synchrony did not change PPS representation. We conclude by proposing a simple, biological-plausible model to explain plasticity in PPS representation after tool-use, which is supported by computational and behavioral data.

## Introduction

Stimuli from different sensory modalities (somatosensation, vision, audition) occurring on or close to the body are integrated in order to provide a multisensory representation of the space where the body physically interacts with objects in the environment, that is the peripersonal space (PPS). Premotor and posterior parietal areas in the monkey brain contain bimodal (visuo-tactile and auditory-tactile) or trimodal (visuo-audio-tactile) neurons with a tactile receptive field centered on a specific body part (head, face neck, trunk or shoulders) and a visual (Rizzolatti et al., 1981; Graziano et al., 1994, 1997; Duhamel et al., 1998) and/or an auditory (Graziano et al., 1999; Schlack et al., 2005) receptive field overlapping the tactile RF and extending to the space around it for a limited distance (usually about 30 cm).

Analogous forms of multisensory responses specifically for the space around the body have been repeatedly shown also in humans and in brain areas homologous to those where PPS neurons have been shown in monkeys (Bremmer et al., 2001; Makin et al., 2007; Gentile et al., 2011; Serino et al., 2011; Brozzoli et al., 2012).

Our group has recently developed a new audio-tactile interaction task to measure the extension of PPS representation (Canzoneri et al., 2012, 2013a,b; Teneggi et al., 2013). Briefly, participants performed a speeded tactile detection task on a body part while concurrent task-irrelevant sounds approached toward, or receded from, the stimulated body part. We found that sounds speeded up the tactile reaction time (RT) only when they were administered within a limited distance from the hand, i.e., within the boundaries of PPS representation (see also Serino et al., 2007, 2011). Using dynamic sounds, we were able to calculate the critical distance where sounds affected tactile RT along a continuum between near and far space, thus estimating the boundaries of PPS.

A critical property of PPS representation is that it is dynamically modified through experience. Using a tool to reach objects in the far space extends the boundaries of PPS representation. In monkeys, Iriki et al. (1996) showed that hand-centered visual RFs of neurons located in the intraparietal sulcus extended after a training period of using a rake to retrieve pieces of food placed at a distance. In humans, neuropsychological (Farnè and Làdavas, 2000; Maravita et al., 2001) and psychophysical (Holmes et al., 2004; Maravita and Iriki, 2004; Serino et al., 2007) studies demonstrated that, after using a tool, crossmodal interactions between tactile stimuli at the hand and visual or auditory stimuli in the far space—i.e., at the location where the tool has been used—increase, suggesting extension of PPS representation. Taken together, these findings suggest that the extent of PPS representation is dynamically shaped depending on experience, extending the action possibilities of the body over its structural limits (see Maravita and Iriki, 2004; Gallese and Sinigaglia, 2010; Costantini et al., 2011). Although the aforementioned conclusion is widely accepted (but see Holmes et al., 2007; Holmes, 2012), the mechanism underlying PPS plasticity after tool-use is currently unknown.

We have recently developed a computational neural network model describing visual-tactile representation of PPS around the hand (Magosso et al., 2010a,b). The model was able to reproduce the characteristic behavior of PPS neurons, namely that they respond to tactile stimuli and to visual stimuli presented near, but not far, from the body. The model was also able to reproduce the extension of PPS due to tool-use (Magosso et al., 2010b): after a simulated tool-use training, the model PPS neurons respond to stimuli presented in the far space. Interestingly, the model also generates a testable prediction to explain how such plasticity in PPS representation may be generated. According to the model, PPS extension after tool-use does not depend on the tool itself, but it is a consequence of pairing tactile stimulation at the hand location (via the tool handle) with synchronized visual stimuli occurring in the far space (at the functional part of tool). Thus, it is possible to predict that simple presenting tactile near stimuli and synchronous visual (or auditory) far stimuli, independently from any tool use, would be sufficient to extend PPS representation. On the contrary, no PPS extension is predicted in case of asynchronous tactile and visual (or auditory) stimulation.

The aim of the present study is to empirically test, in both computational and behavioral experiments, the aforementioned prediction generated by our computational model. In order to measure behaviorally the extension of PPS representation, we used the audio-tactile paradigm we recently developed (Canzoneri et al., 2012; Teneggi et al., 2013). However, the PPS computational model originally described in Magosso et al. (2010a) focused on visuo-tactile interaction. Thus, in the first part of the present study we also present a new audio-tactile version of the PPS computational model, simulating audio-tactile, instead of visual-tactile, interaction in the space around the hand. Indeed, accounting for the characteristic of the auditory system in the model is important to generate solid and reliable predictions to be tested behaviorally using the audio-tactile interaction paradigm. In a first simulation experiment (Section The Computational Study), the model was then applied to simulate the effects on PPS representation of an audio-tactile training that consists in administering tactile stimuli on the hand together with synchronous auditory stimuli in the far space. This training would simulate the sensory inputs gathered in case of using a tool to interact with objects in the far space. As a control condition, we also tested the effects of an asynchronous audio-tactile training, in which auditory and tactile stimuli were not correlated in time. We predict that the synchronous, but not the asynchronous training, would extend PPS representation, that is it would make PPS neurons responding to far auditory stimuli at the end of the training, differently than before the training.

In an *in vivo* experiment (Section The Behavioral Study), we then tested behaviorally the model's prediction that a tactile and far-auditory synchronous stimulation extends PPS representation in humans, even without any use of the tool. To this aim, we assessed PPS representation in a group of volunteers before and after an audio-tactile stimulation training. During the training, subjects received a tactile stimulus at the hand while a concurrent auditory stimulus was synchronously presented in the far space (at 1 m from the hand). As a control condition, participants' PPS representation was also measured before and after an asynchronous training consisting in tactile stimuli delivered at the hand and auditory far stimuli, with a randomized temporal delay between the two. To measure the extension of PPS before and after auditory-tactile stimulation, we took advantage from our new audio-tactile interaction paradigm (Canzoneri et al., 2012; Teneggi et al., 2013), which allows estimating the critical distance where sounds speeded up tactile reaction time as a proxy of PPS boundaries. That distance was compared before and after the synchronous and asynchronous trainings. Following the model prediction, we hypothesized that after the synchronous, and not after the asynchronous audio-tactile training, PPS boundaries would extend toward the far space, as it happens following a classic tool-use training (see Canzoneri et al., 2013a).

## The computational study

### Neural network description

Here, we briefly introduce a neural network model representing audio-tactile interaction in PPS. This model is a modified version of the model described in Magosso et al. (2010b) (see also Magosso et al., 2010a) focusing on visual-tactile interaction in the space around the hand. Modifications were necessary to replace the visual modality with the auditory modality, requiring adjustment of nodes and connections parameters within the network. In particular, an important point to be accounted for is the lower spatial resolution of the auditory system compared with the visual one. In this section, qualitative model description is presented. A quantitative description of the model with all equations, parameter values and simulation details can be found in the Supplementary Material.

**Figure 1:**
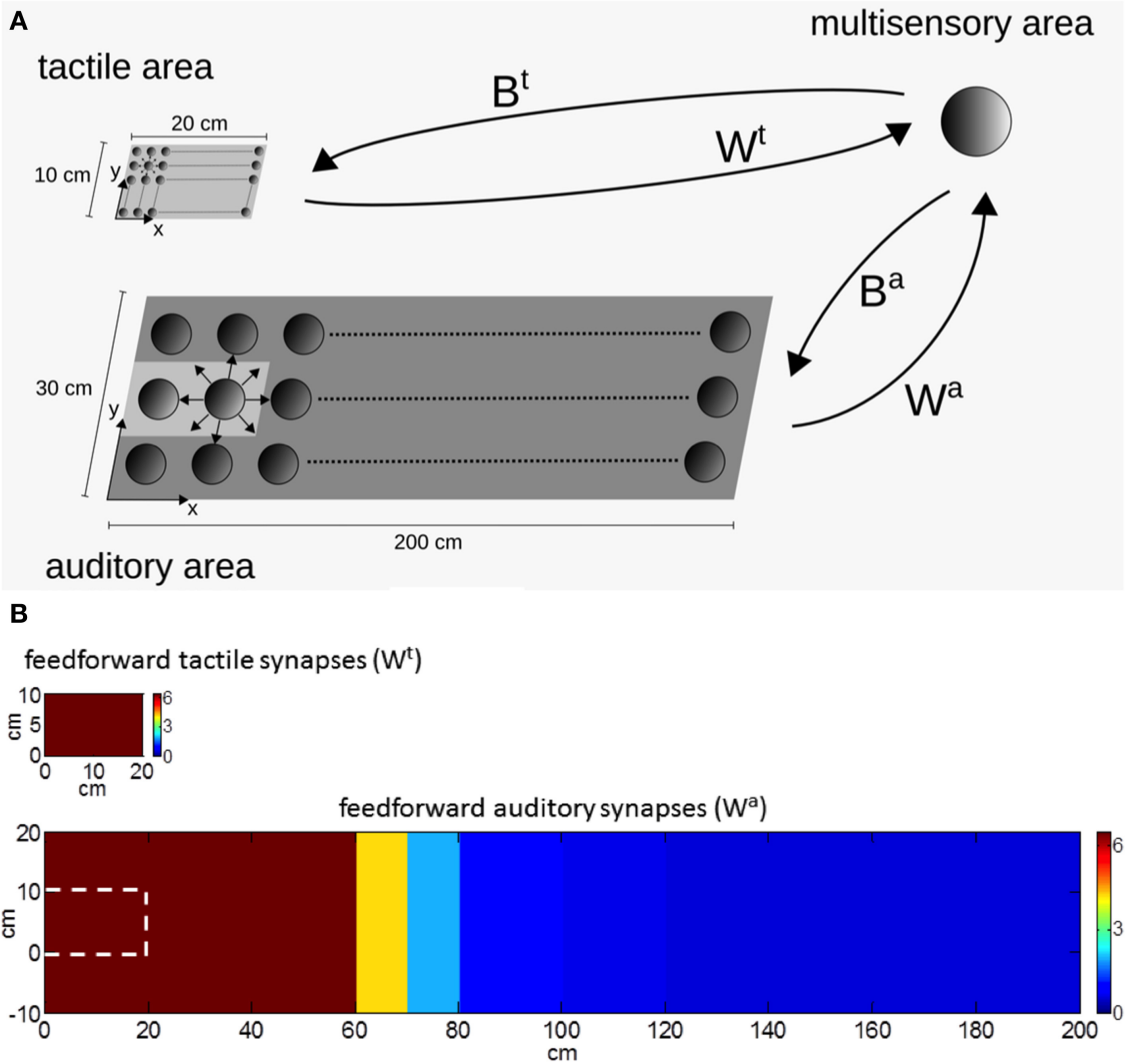
**(A)** Schematic diagram of the audio-tactile network. The network includes a unisensory tactile area, a unisensory auditory area and a multisensory area. Each filled circle represents a neuron. Smaller cycles mean neurons having smaller receptive fields. The unisensory areas and the multisensory area communicate via reciprocal synapses: W^t^, W^a^ are feedforward synapses from the unisensory tactile and auditory neurons, respectively, to the multisensory neuron; B^t^, B^a^ are the feedback synapses from the multisensory neuron to the unisensory tactile and auditory neurons, respectively. **(B)** Pattern of the tactile and auditory feedforward synapses (W^t^, W^a^) in basal conditions (i.e., before network training). The white dashed line, in the image displaying auditory synapses, represents the region corresponding to the hand.

Briefly, the model includes two unisensory areas (tactile and auditory) communicating—via synaptic connections—with a third multisensory (audio-tactile) area (Figure 1A).

The tactile unisensory area contains a matrix of neurons mapping a surface of 20 cm × 10 cm, approximately representing the surface of the whole hand. The auditory unisensory area contains a matrix of neurons mapping a space of 200 cm × 30 cm on and around the hand, where stimuli potentially interacting with the hand can occur. Each unisensory neuron has its own receptive field (RF): in both areas, neuron RFs are in hand-centered coordinates. Since the auditory system is characterized by a low spatial resolution, the auditory neurons have been assigned large RFs. Additionally, unisensory neurons within each area reciprocally interact via lateral synapses arranged according to a Mexican hat disposition (near excitation, far inhibition). Each unisensory area may receive an external spatially localized (tactile or auditory) stimulus: the resulting activation in the area is determined by the neuron RFs joined with the action of lateral synapses. In particular, due to the large auditory RFs, an auditory stimulus induces a wide activation in the auditory area.

The two unisensory areas send feedforward excitatory synapses to the downstream multisensory area, which is devoted to the bimodal (audio-tactile) representation of the PPS around the hand. For sake of simplicity, only one multisensory neuron is considered (see Magosso et al., 2010b). The feedforward synapses from the tactile neurons to the multisensory one have all the same value (i.e., independent from the location of the tactile neuron RF, see Figure 1B). On the contrary, the feedforward synapses from the auditory neurons decrease their value as a function of the distance of the auditory RF from the hand (Figure 1B), so that, auditory neurons having RFs on or near the hand send stronger synapses to the multisensory area than auditory neurons with RF placed far from the hand. The feedforward tactile and auditory synapses—combined with the RFs of the unisensory neurons—shape the tactile and auditory RF of the multisensory neuron.

The multisensory neuron, in turn, sends feedback excitatory synapses to the unisensory upstream areas; the feedback synapses have been given the same arrangement as the feedforward ones. Via the feedback synapses, activities in the unisensory areas may influence reciprocally, that is, in case of a multimodal (audio-tactile) stimulation, the stimulus in one modality (e.g., the auditory one) may affect activation in the other unisensory area (e.g., the tactile one).

Each neuron responds to its input via a temporal dynamics before it settles to a final activation value (maximum neuron activation is 1). Hence, network response to a stimulus develops gradually in time until the new steady-state condition is reached.

**Figure 2:**
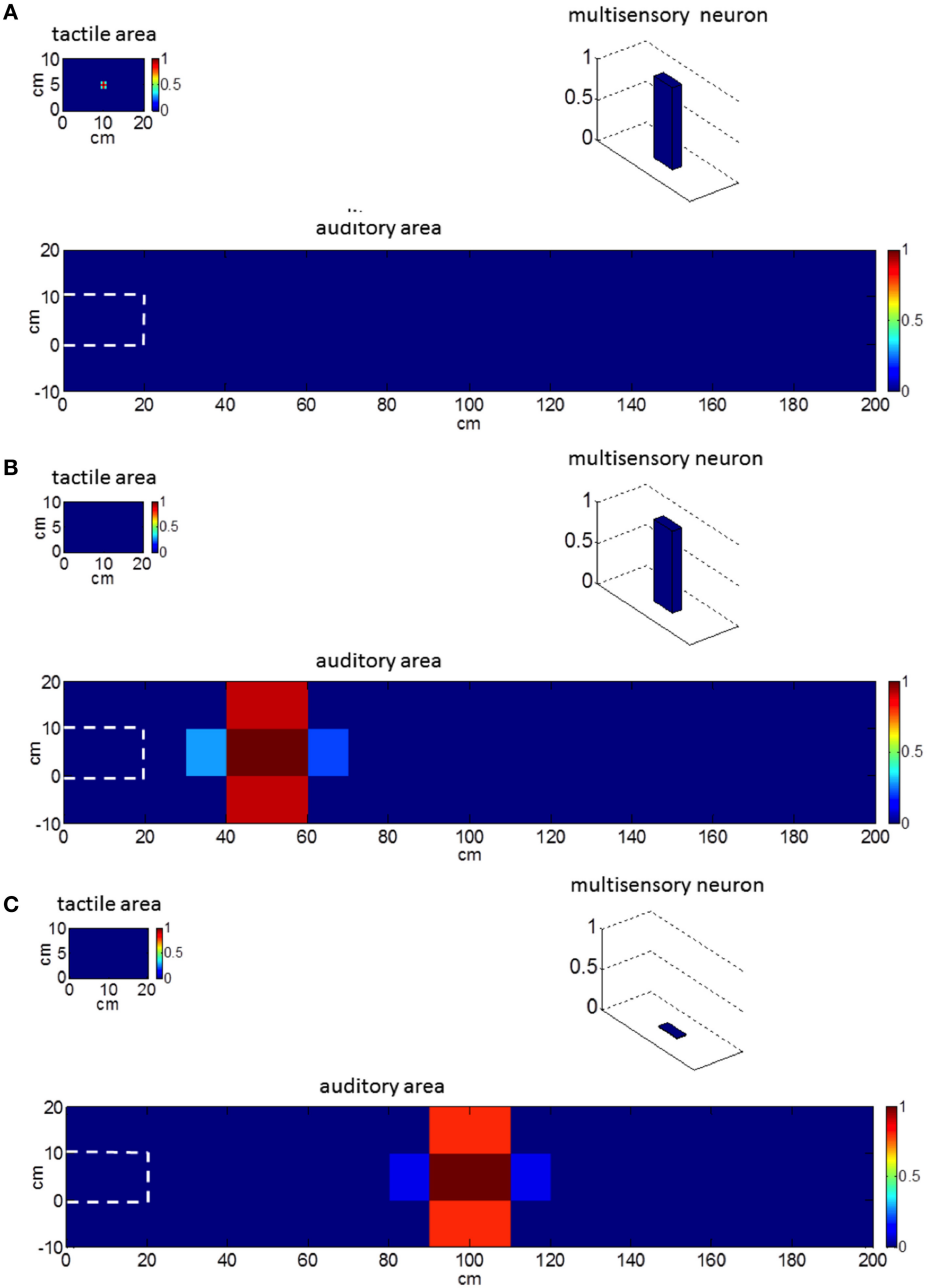
**Network behavior in basal conditions in response to unimodal stimulation**. Plots refer to the final steady-state condition. **(A)** Network response to a tactile stimulus. The multisensory neuron is activated. **(B)** Network response to an auditory stimulus close to the hand (at position *x* = 50 cm, *y* = 5 cm, i.e., at a distance of 30 cm from the hand). The multisensory neuron is activated. **(C)** Network response to an auditory stimulus far from the hand (at position *x* = 100 cm, *y* = 5 cm, i.e., at a distance of 80 cm from the hand). The multisensory neuron is silent. Worth noticing that the external tactile and auditory stimuli have the same spatial extension: the larger activation in the auditory area is the consequence of the bigger auditory RFs. The white dashed line denotes the auditory space on the hand.

Figure 2 exemplifies the behavior of our network in response to unimodal stimulation. A tactile stimulus on the hand (Figure 2A) and an auditory stimulus close to the hand (Figure 2B) activate the corresponding unisensory area as well as the multisensory neuron. On the contrary, an auditory stimulus from the far space (Figure 2C) activates the unisensory neurons but not the multisensory one. Hence, the multisensory neuron in the model mimics the responses of PPS neurons as observed in the posterior parietal areas of the primate brain (Graziano et al., 1999; Schlack et al., 2005), responding to tactile stimuli on the monkey's body and to auditory stimuli presented close to monkey, but not far apart (See Figure 2).

In the last two decades, several models have been proposed to study multisensory integration. Most of these models are based on a Bayesian approach, providing a set of probabilistic rules that can predict perceptual phenomena of cue integration (Knill and Richards, 1996; Ernst and Banks, 2002; Alais and Burr, 2004; Shams et al., 2005). These models, however, do not propose any insight about the underlying neural mechanisms. Other models, on the other hand, have attempted to tackle the neural processes involved in multisensory integration. In particular, Pouget and colleagues proposed an influential computational framework to formalize multisensory integration in the context of reference frames transformation (Pouget and Sejnowski, 1997; Deneve et al., 1999; see Pouget et al., 2002 for a review). Briefly, their networks consist in multiple layers coding unisensory inputs in their original frames of reference, interconnected with different multisensory layers. Multisensory integration occurs in multiple modules within the parietal cortex, where stimuli are coded in intermingled reference frames. These modules project to a set of motor modules, that encode the locations of stimuli in frames of reference specific to the task controlled by each system. Our present model (as well as the previous ones we have proposed; see Magosso et al., 2010a,b) shares some important aspects with Pouget's model, such as the multilayer architecture of the network, the presence of recurrent (feedback and feedforward) connections between the unisensory and multisensory areas, the implementation of unisensory neurons with spatial tuning functions. On the other hand, our models include additional features which are crucial for investigating PPS representation, such as dependence of RF's size on the specific sensory modality, patterns of feedforward and feedback synapses tuned on the specific sensory modality and the distinction between coding of stimuli near and far from a body part.

### Neural network training and testing

#### Audio-tactile training

**Figure 3:**
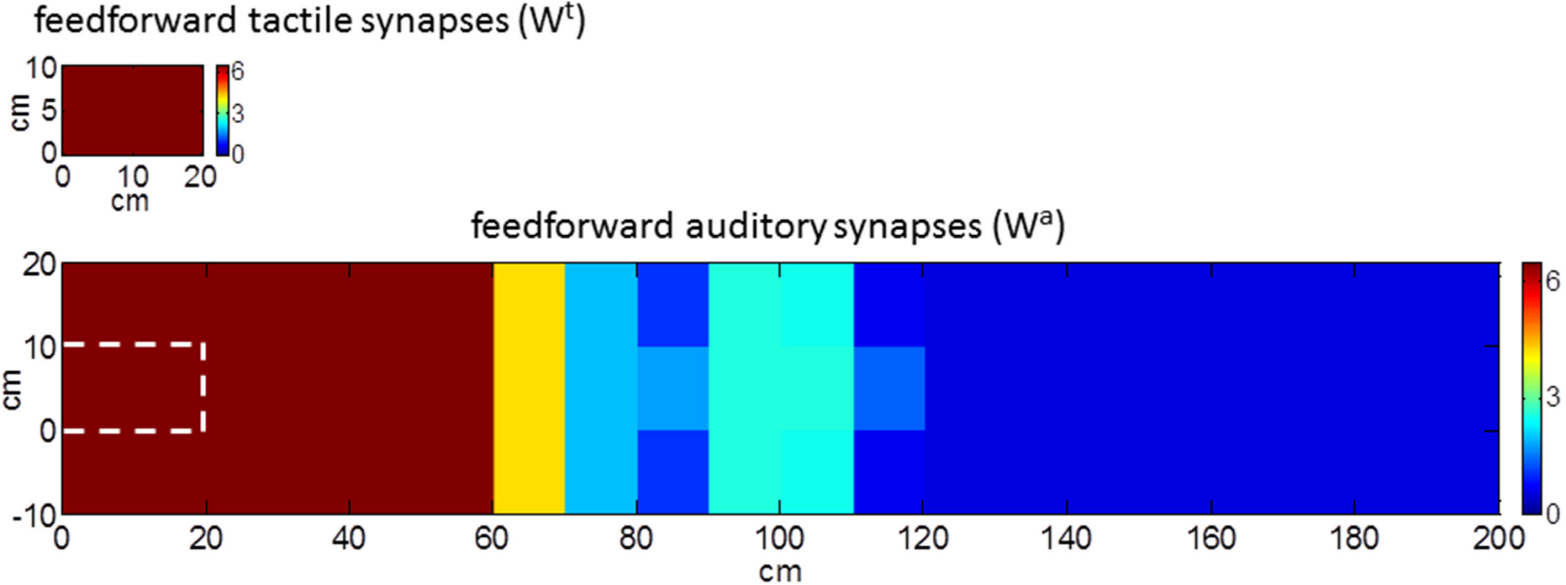
**Pattern of the feedforward synapses after the synchronous audio-tactile training**. After the training (compare with Figure 1B), the auditory synapses placed in the region of space stimulated during the training reinforce. Tactile synapses do not change as feedforward synapses on the hand are assumed already at their maximum value before tool use (i.e., in basal condition).

The model was used to simulate incoming sensory inputs during tool-use training. At the sensory level, the use of a tool produces tactile stimulation on the hand, transmitted by the tool handle, paired with simultaneous visual and/or auditory information from the space where the tool is actively used (i.e., far space). Hence, tool-use training was mimicked by applying a tactile stimulus and a synchronous auditory stimulus as inputs to the network. Tactile stimulation was replicated as a localized tactile input on the hand able to activate both the tactile area and the multisensory area (as in Figure 2A). The auditory stimulation was replicated as an auditory input applied far from the hand at position *x* = 100 cm and *y* = 5 cm (as in Figure 2C). The two stimuli had the same duration and were applied simultaneously. This network stimulation replicates the sensory information input that would occur during the use of a long tool (about 1 m long) to functionally interact with the far space position (*x* = 100 cm). The lack of visual information (as in the present model) may correspond to the case of a subject performing the training in blindfolded conditions (see e.g., Canzoneri et al., 2013a). During the application of the stimuli, the feedforward synapses linking the unisensory neurons with the multisensory one modified according to a Hebbian-like rule. The rule includes a potentiation factor—i.e., synaptic weight increases in the presence of temporally correlated activities of the pre-synaptic and post-synaptic neurons, up to a maximum saturation value—and a forgetting factor—i.e., the reinforced synapsis loses its weight in case of uncorrelated activities. The synchronous audio-tactile stimulation activates the tactile neurons and the multisensory one (due to projections from the tactile area) and produces the simultaneous activation of the auditory neurons with RF placed far from the hand (due to the far auditory stimulus). As a result of the Hebbian mechanism, the synapses linking the auditory neurons having RFs in the far space with the multisensory neuron—which are originally weak (see Figure 1B)—reinforces, due to the simultaneous activation of the pre-synaptic auditory neurons and the post-synaptic multisensory neuron. Figure 3 shows the pattern of the feedforward synapses after a training phase consisting in 30 presentations of synchronous audio-tactile stimulation: feedforward synapses from the auditory to the multisensory neuron strengthened significantly (compare with Figure 1B) in the portion of the auditory area stimulated during the training, i.e., far space. Tactile feedforward synapses did not change, as they are set at their maximal value already in basal (pre-training) conditions.

The network training was replicated in case of an asynchronous audio-tactile stimulation (i.e., control condition). The two stimuli were delivered to the network with a variable, non-null stimulus onset asynchrony (SOA), so that they were randomly partially superimposed or completely separated. This kind of stimulation replicates sensory information stream that may occur in activities not involving the use of a tool, whereby tactile stimulus (due to object manipulation with hands) may be only partially and randomly correlated with far auditory stimuli. In this conditions, the potentiation factor in the Hebbian learning rule is counterbalanced by the forgetting factor: at the end of the training (30 presentations of the asynchronous stimulation), auditory feedforward synapses exhibited only negligible modifications compared to pre-training conditions.

#### Testing the effects of audio-tactile training on PPS representation

In a series of simulations, we evaluated the effect of the training on PPS representation in the model. To this aim, we first assessed how the training affected the response of the multisensory neuron to an unimodal auditory stimulus placed at different positions in space, i.e., at different distances from the hand. The intensity of the auditory stimulus was randomly varied to generate variability in network response. For each position, the stimulus was applied 30 times to the untrained network and to the trained network; for each simulation, the final activation of the multisensory neuron was computed. In this way, we evaluated how the auditory RF of the multisensory area was modified by the training.

Then, in order to directly compare the results obtained in simulation experiments with those obtained in *in vivo* behavioral experiments on human subjects, we also presented the neural network with audio-tactile stimuli, to reproduce the paradigm recently developed to estimate the boundaries of PPS (Canzoneri et al., 2012, 2013a,b; Teneggi et al., 2013). In that paradigm, tactile RT on a body part is evaluated while a sound is simultaneously presented at different distances from the body part: the critical distance where the sounds speed up tactile RT is considered as a proxy of PPS boundaries. To replicate this experiment, the network—both before training and after training—was fed with a tactile stimulus on the hand (able by itself to activate the multisensory neuron, as in Figure 2A) and a simultaneous auditory stimulus applied at different distances from the hand. For each of the 15 tested sound distances (between 140 and 0 cm from the hand), the stimuli were applied 30 times to the untrained network and to the trained network. For each simulation, we computed the time necessary for the tactile area to reach 90% of its final activation as a measure representative of tactile RT in the network. The influence the auditory stimulation may exert on the tactile response (measured in terms of reaction time) is mediated by the multisensory area via the feedback synapses. When the auditory stimulus is not able to trigger the multisensory neuron, unimodal tactile response is unaffected by auditory stimulation, When the auditory stimulus is able to trigger the multisensory neuron, it contributes to speed up tactile unimodal activation. Therefore, tactile RT for sounds at different distances depends on the extension of the auditory RF of the multisensory area.

### Results

**Figure 4:**
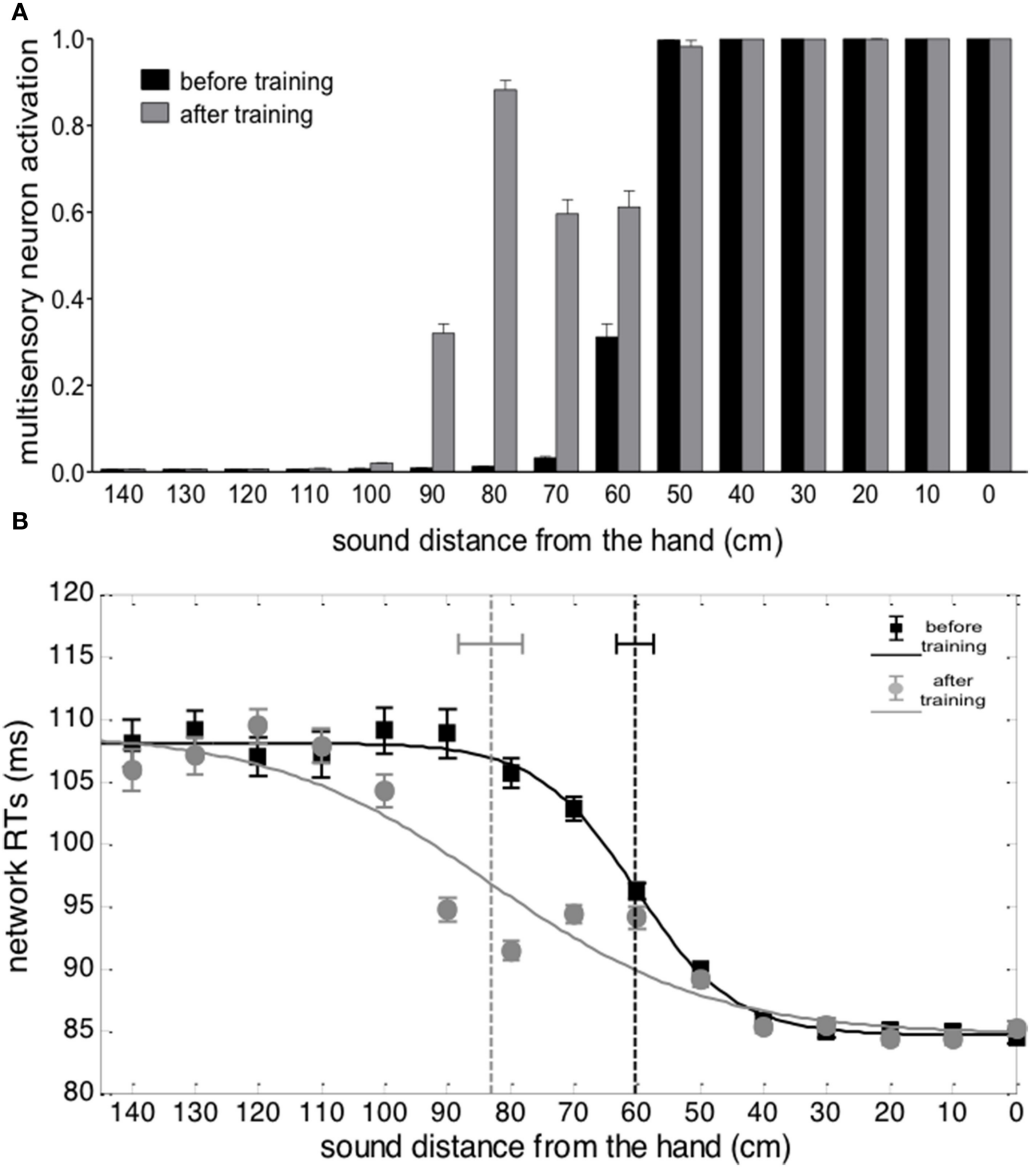
**Effects of audio-tactile synchronous training on PPS representation**. **(A)** Activation of the multisensory neuron to an auditory stimulus at different distances from the hand, before training (black bar) and after training (gray bar). The stimulus intensity was affected by a random noise. For each position, the stimulus was presented 30 times: each bar depicts the mean value of the multisensory neuron activation ± SEM. **(B)** Network tactile RT with the sound at different distances, before training (black square symbols) and after training (gray circle symbols), and the fitting sigmoidal curves (continuous lines). The auditory and tactile stimuli were simultaneous and their intensity was affected by noise. For each position, 30 simulations were performed. Symbols denote the mean value of network tactile RT ± SEM. The dashed vertical lines denotes the estimated central point of the sigmoidal function, with the 95% CI of the estimated parameter.

Responses of the multisensory neuron to unimodal auditory stimuli are reported in Figure 4A. Before the training, the multisensory neuron responded to auditory stimuli located at a limited distance from the hand (within 50–60 cm from the hand). After training in the synchronous condition, the multisensory neuron responded also to stimuli presented farther apart (up to ≈90 cm), showing that the synchronous audio-tactile training has produced an extension of PPS representation. This ensues from modification of the auditory feedforward synapses that causes an enlargement of the auditory RF of the multisensory neuron to include the far space (where the auditory stimulus was localized during the training, Figure 3). Such model result is consistent with single-cell recordings in monkey, showing that the visual hand-centered RF of parietal multisensory neurons extends to cover the far space after a tool-use training period (Iriki et al., 1996; Hihara et al., 2006).

Figure 4B shows network responses to audio-tactile stimuli. Tactile RTs obtained at the different sound positions are shown before training and after the synchronous training. In both cases, tactile RTs became lower as the distance of the auditory stimulus from the hand decreased. However, before training, the speeding effect of the sound on touch occurred for sounds applied approximately within 60 cm from the hand; conversely, after training, RT was speeded up even by sounds at farther distances (70, 80, and 90 cm). These patterns of tactile RTs can be explained as follows. When the auditory stimulus falls within the auditory RF of the multisensory neuron, it participates—together with the tactile stimulus—to the activation of the multisensory neuron. In this condition, the multisensory neuron reaches its maximum activation level more quickly and speeds up—via the feedback synapses—the activation in the unisensory tactile area, decreasing the tactile RT of the network. This speeding effect does not occur when the auditory stimulus is not able to trigger the multisensory neuron (i.e., when it falls outside the auditory RF of the multisensory neuron). Accordingly, the expansion of the auditory RF of the multimodal neuron after training (see Figures 3, 4A), gives rise to a decrease of tactile RT by means of auditory stimuli applied at farther locations in space then before training. The network's tactile RT's at the different sound positions were fitted with a sigmoidal function (separately, before training and after training): the sigmoid central point estimated via the fitting procedure was taken as an index of the critical distance at which the sound started affecting RT, i.e., where the boundary of PPS was located (see Supplementary Material for more details on the fitting procedure). The model predicts that after training the central point of the sigmoid was shifted at a greater distance from the hand than before training (CI_95%_ = 78.1 ÷ 88.2 cm after training vs. CI_95%_ = 57.4 ÷ 63.2 cm before training). It is worth noticing that results obtained in Figure 4 depend on all mechanisms included in the network: large RFs of auditory neurons, summation of tactile and auditory inputs at multisensory level, synaptic Hebbian rule, feedback synapses from multisensory to unisensory areas.

**Figure 5:**
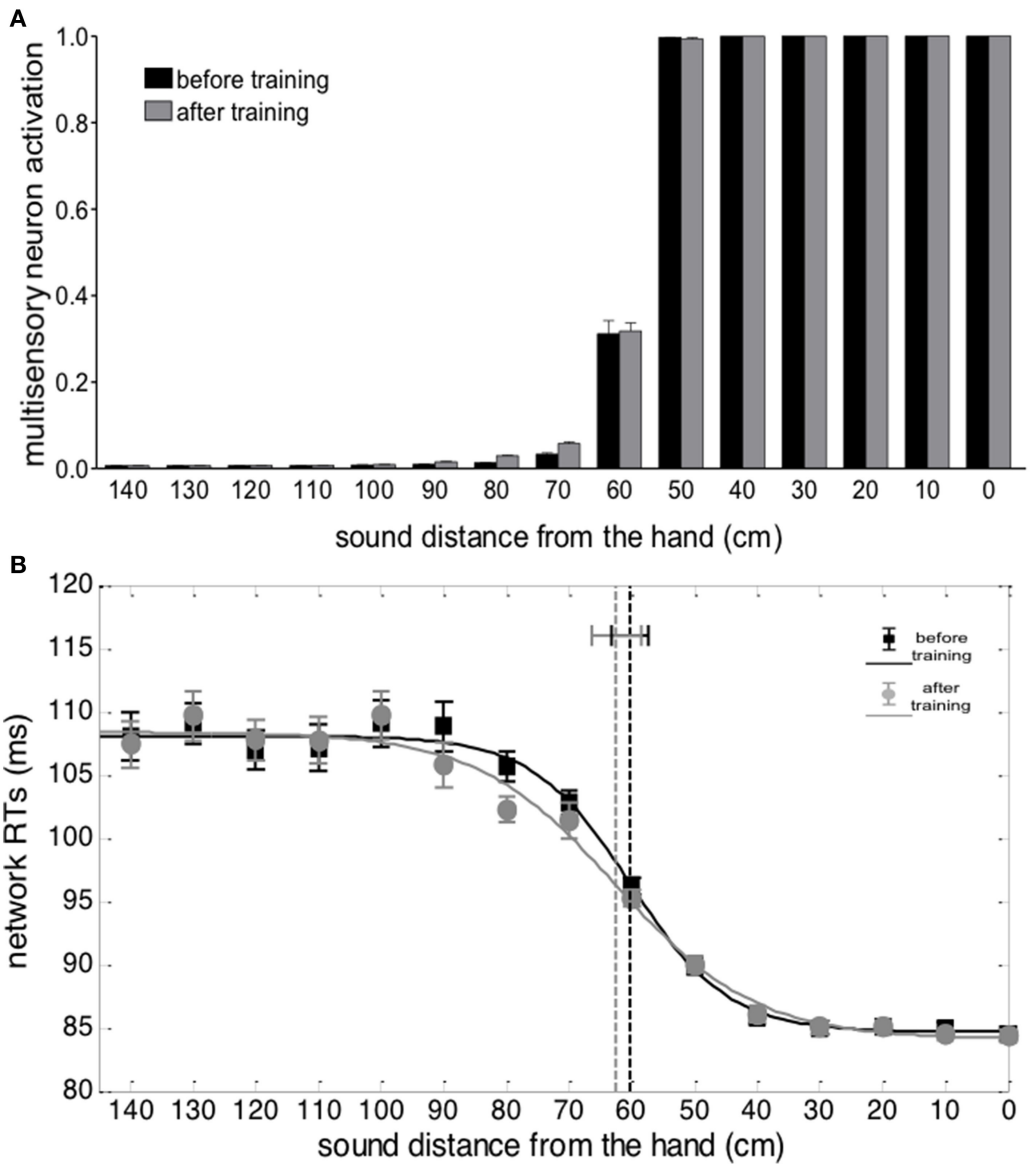
**Effects of audio-tactile asynchronous training on PPS representation**. Data are reported as for Figure 4. Asynchronous training did not produce any significant change compared to before training conditions.

Figure 5 shows the results from auditory and audio-tactile stimulation provided before and after the training in the asynchronous condition. No significant difference in the network response was observed between the experiments run before and after the asynchronous training (Figures 5A,B). In particular, the sigmoidal function fitting the relationship between network RT and sound distance did not exhibit a significant modification of its central point after training vs. before training (CI_95%_ = 58.6 ÷ 66.4 cm after training vs. CI_95%_ = 57.4 ÷ 63.2 cm before training), suggesting no modification in PPS representation. This null effect is in line with the absence of any significant change in the feedforward synapses from the auditory and the multisensory areas of the network.

In conclusion, the model predicts an expansion of PPS boundaries as a consequence of a synchronous application of a tactile stimulus on the hand and an auditory stimulus far from the hand, simulating incoming sensory information when using a tool to interact with the far space. Hence, the model generates the novel hypothesis that neither the physical presence nor active use of a tool is necessary to induce an extension of PPS expansion. Rather, the model is able to capture contingencies between far auditory stimulation and tactile stimulation due to their temporal synchrony, as normally conveyed in case of tool use. Thanks to synaptic plasticity mechanisms in the model, based on Hebbian-like learning rules, such contingency is sufficient to extend PPS representation. This hypothesis has been tested *in vivo* in the behavioral experiment, taking advantage of the audio-tactile behavioral task.

## The behavioral study

### Participants

Sixteen healthy subjects (12 females, age ranging between 23 and 26 years) participated in the study. All subjects were right-handed and had normal hearing and touch. All subjects, students at the University of Bologna, gave their informed consent to participate in the study, which was performed in accordance with the Declaration of Helsinki and approved by the Ethical Commission of the Department of Psychology, University of Bologna.

### Materials and procedures

#### Measuring PPS representation

**Figure 6:**
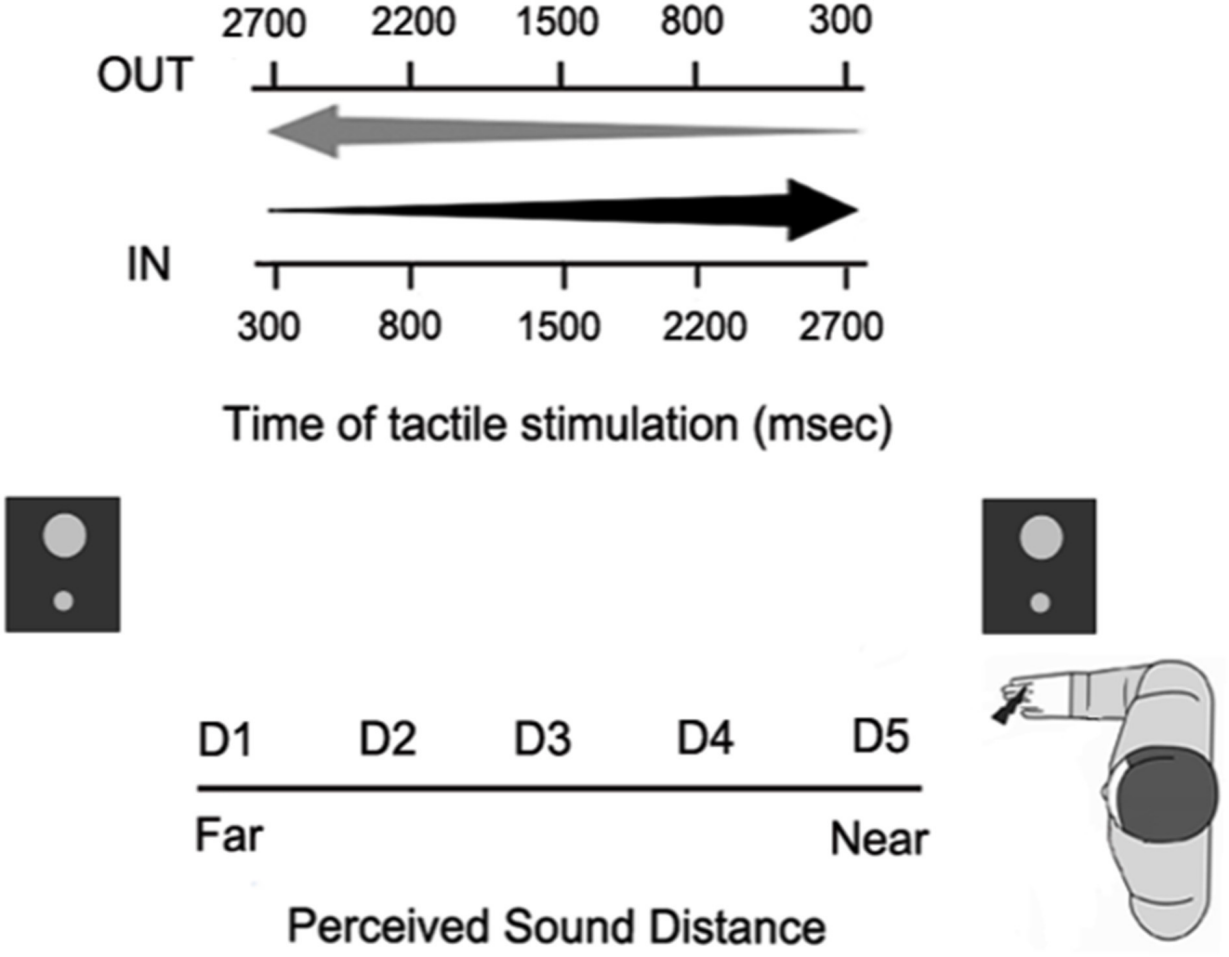
**Paradigm of the audio-tactile interaction task used in the behavioral experiments**. Participants responded to a tactile stimulus on their hand, while task-irrelevant sounds either approached toward (IN sounds) or receded from (OUT sounds) their hand. On each trial, the tactile stimulus was delivered at one out of five possible different delays from sound onset, so that it was processed when the sound was perceived at a different distance from the subject's body (from D1, very far, to D5, very close).

In order to assess the extension of PPS representation around the hand, we used an audio-tactile interaction task as in Canzoneri et al. (2013a) and Teneggi et al. (2013), (see Figure 6). During the experiment, subjects were comfortably seated beside a table, which the audiotactile apparatus was mounted on. This consisted of (a) two loudspeakers (hidden from view), one placed close to the participants' right hand (at ~5 cm), the other one placed at a distance of ~100 cm from the near loudspeaker, thus far from the participant; and (b) a constant current electrical stimulator (DS7A, Digitimer, Hertfordshire, United Kingdom), controlling a pair of neurological electrodes (Neuroline, Ambu, Ballerup, Denmark), attached on the participant's right hand. Auditory stimuli were samples of pink noise of 3000 ms duration, whose intensity was manipulated in order to generate two kinds of sounds: IN sounds gave the impression of a sound source moving from the far to the near loudspeaker, i.e., toward the subject; OUT sounds gave the impression of a sound source moving in the opposite direction, i.e., receding from the subject. During each trial, either an IN or an OUT sound was presented, while, in 77% of the trials, subjects also received a tactile stimulus on their right hand. The remaining trials were catch trials with auditory stimulation only. Subjects were asked to respond vocally as fast as possible to the tactile target, when present, trying to ignore concurrent sounds. Tactile RTs were recorded via a voice-activated relays. A custom-made software (C.I.R.O.) was used to control stimuli administration and responses recording.

The tactile stimulus was delivered at different temporal delays from the onset of the auditory stimulus, so that touch was processed when the sound was perceived at different locations with respect to the subject's body. Five different delays from T1 to T5 (300, 800, 1500, 2200, 2700 ms) were used both for approaching and receding sounds. A sound localization experiment, published in Canzoneri et al. (2012), showed that at every temporal delay, sounds were perceived in a different position of space with respect to the stimulated body part. Given the equivalent segmentation of the different temporal delays for the two kinds of sounds, there was a spatial correspondence between the perceived position of IN and OUT sounds at T1 IN and T5 OUT (farthest distance from the body) and at T2 IN and T4 OUT (far distance), T3 IN and T3 OUT (intermediate distance), T4 IN and T2 OUT (close distance), T5 IN and T1 OUT (closest distance). Therefore, the corresponding temporal delays were recoded in terms of perceived sound distance from D1 – farthest distance—to D5 – closest distance.

#### Synchronous audio-tactile training

During the training participants were blindfolded and sat down with their right arm resting palm down on a table beside them. Participants received different trains of audio-tactile stimuli. Each train consisted of 10 tactile stimuli and 10 auditory stimuli, synchronously presented. Tactile stimulation was administered through two solenoids applied at the tip of the right index finger (M and E Solve, Rochester, UK). The auditory stimulation consisted in an ecologic sound (the tapping of a pencil on a table) previously recorded. In this way, audio and tactile stimulation used for the training (i.e., ecological sounds and vibro-tactile stimulation) was different from that used for measuring PPS representation (i.e., pink noise and electrocutaneous stimulation). The sound was presented through two loudspeakers, placed on the table at a distance of ≈100 cm from participants' hand. A PC running C.I.R.O. software (www.cnc.unibo.psice.unibo/ciro) was used to control the presentation of the stimuli. During the training participants received 23 trains of stimuli, interleaved with 22 interstimulus intervals. Each train lasted 5000 ms. The inter stimuli interval randomly varied between 4000 and 6000 ms. In order to control for participants' attention during the training, five auditory stimuli (a “beep”) were randomly presented during the inter stimuli intervals. Participants were asked to respond to the “beep” stimuli by tapping their foot on the floor. Each training session lasted around 5 min.

#### Asynchronous auditory training

The same auditory and tactile stimuli used for the synchronous training were used for the asynchronous training, but in the latter condition auditory and tactile stimuli were presented with a systematic temporal delay of 500 ms between them. This way, neither a spatial (tactile stimulus at the hand and auditory stimulus in the far space) nor a temporal coincidence was present between auditory and tactile events.

#### Design

Participants performed both the Synchronous and the Asynchronous auditory training in two different days. In each day of testing, we measured PPS representation before and after the training sessions. In Day 1, before tool-use, participants performed the audio-tactile interaction task to assess PPS representation in a baseline condition. Then, they performed two sessions of the synchronous auditory training. Each session was intermingled with an assessment session, consisting of one block of the audio-tactile interaction task. On a different day, participants underwent the same procedure with the asynchronous training. The order of synchronous or asynchronous training was counterbalanced between subjects.

### Results

Participants were extremely accurate in responding to the beep stimuli during both the synchronous and asynchronous training, meaning that they paid attention during the training (mean accuracy 98.6 and 98.5%). In order to compare the extent of PPS representation before and after the training in the synchronous and asynchronous conditions, we analyzed RTs to the tactile target as a function of the different perceived distance of the approaching and receding sounds at the time of tactile stimulation. To this aim, we run a mixed ANOVA on tactile RTs with Training condition (Synchronous, Asynchronous), Session (Before Training, After Training), Sound (IN, OUT) and Distance (from D1 to D5) as within subjects factors, and Order of training (Synchronous–Asynchronous; Asynchronous–Synchronous) as between subject and factor. RTs exceeding more than two standard deviations from the mean RT, calculated for each subject in each condition, were trimmed from the analysis.

Results showed a significant Training × Distance interaction [*F*_(4, 56)_ = 2.66, *p* < 0.05; η^2^ = 0.15]. In order to explore how the different training (Synchronous, Asynchronous) affected participants' responses in the audio-tactile interaction task at different distances, we then conducted two separate ANOVAs, one for each training.

**Figure 7:**
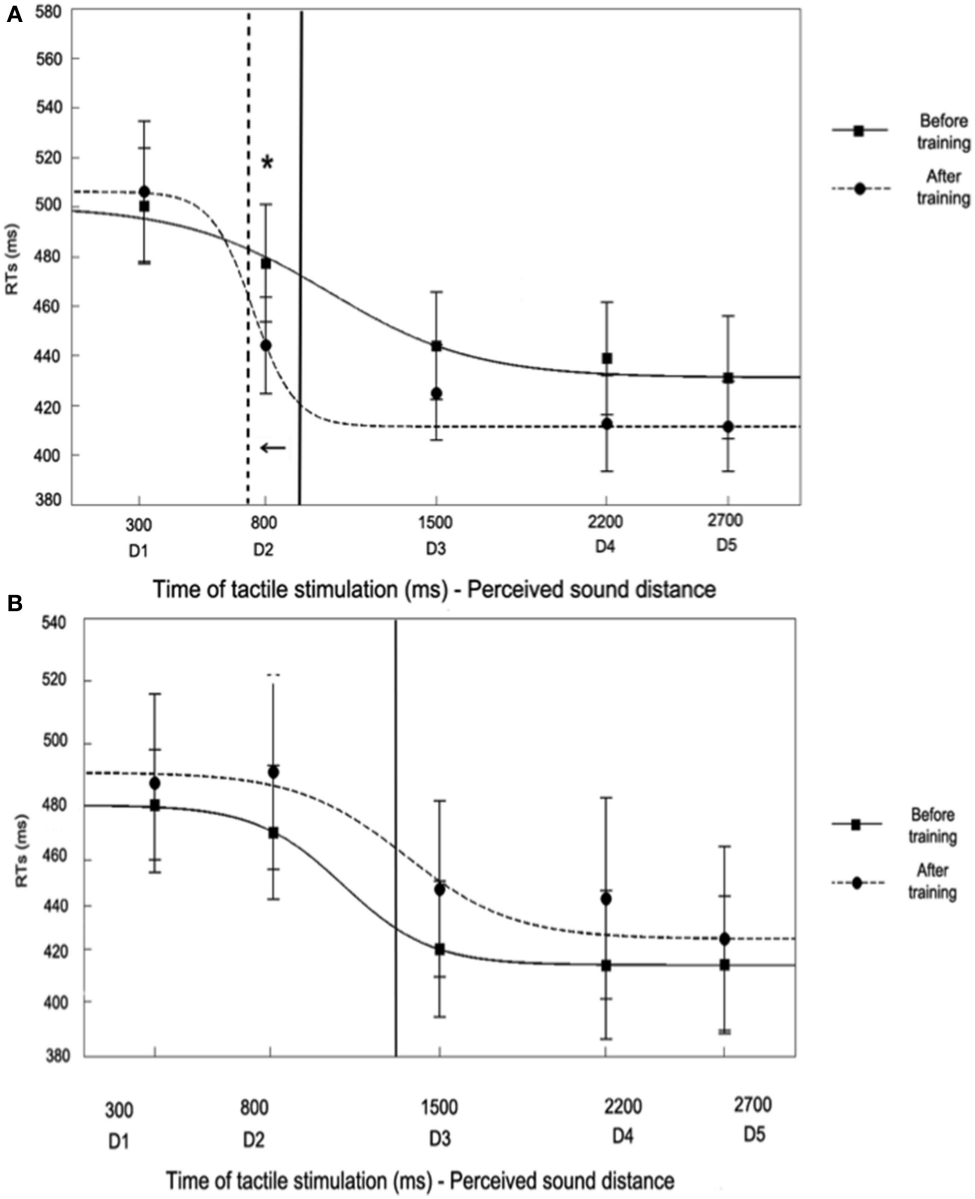
**Audio tactile interaction task results (for the IN sound) before and after the training the Synchronous condition (A) and Asynchronous (B) conditions**. Figures report mean RTs (and S.E.M.) to tactile hand stimulation at different perceived sound distances from D1 – farthest—to D5 – closest—(corresponding to different times of tactile stimulus delivery), Before Training (filled line) and After Training (hatched line). RTs at the different time intervals were also fitted to a sigmoidal function described by the following equation:
$$y(x)=\frac{y_{min}+y_{max}\;\cdot \;e^{(x\;-\;x_{c})/b}}{1+e^{(x\;-\;x_{c})/b}},$$
where *x* represents the timing of touch delivery in ms, *y* the reaction time, *y*_*min*_ and *y*_*max*_ the lower and upper saturation levels of the sigmoid, *x_*c*_* the value of the abscissa at the central point of the sigmoid (i.e., the value of *x* at which *y* = (*y*_*min*_ + *y*_*max*_)/2), and *b* establishes the slope of the sigmoid at the central point. The sigmoid central point was computed as a measure of the temporal delay, i.e., the distance, at which sounds start to affect RTs and was analyzed in order to quantify PPS boundaries. In the Synchronous condition **(A)**, the sigmoid central point was higher in the Before Training (1055 ms) as compared to the After Training condition (745 ms), meaning that PPS boundaries were localized as farther from the body after the training.

For the Synchronous training condition, the ANOVA conducted on RTs with Session (Before Training, After Training), Sound (IN, OUT) and Distance (from D1 to D5) as within subjects factors showed a significant three-way interaction [*F*_(4, 60)_ = 2.57, *p* < 0.05, η^2^ = 0.15]. We have repeatedly shown that the present task is especially sensitive to approaching as compared to receding sounds (Canzoneri et al., 2012; Teneggi et al., 2013), therefore here we focused on results concerning the IN sounds only (OUT sounds data are reported in Table 1). Before Training, for the IN sound, the function describing the relationship between tactile RTs and the perceived position of sound in space showed that tactile RTs progressively sped up as the perceived sounds' distance from the body decreased (See Figure 7A).

**Table 1:** **Audio tactile interaction task results for the OUT sound, both for Synchronous and Asynchronous Conditions**.

		**D1**	**D2**	**D3**	**D4**	**D5**
Synchronous Condition	Before Training	505 ms ± 27	474 ms ± 24	460 ms ± 25	476 ms ± 25	477 ms ± 26
	After Training	466 ms ± 26	451 ms ± 23	447 ms ± 23	457 ms ± 23	445 ms ± 23
Asynchronous Condition	Before Training	466 ms ± 22	443 ms ± 22	426 ms ± 21	447 ms ± 20	449 ms ± 23
	After Training	485 ms ± 27	471 ms ± 31	457 ms ± 32	474 ms ± 30	451 ms ± 29

Mean (and S.E.M.) RTs at different perceived sound distances from D1 – farthest—to D5 – closest—(corresponding to different times of tactile stimulus delivery), Before and After Training.

In particular, RTs at D1 (mean RTs ± S.E.M; 517 ms ± 23) and D2 (497 ms ± 24)—when sounds were perceived far from the body—were significantly slower as compared to D3 (464 ms ± 22), D4 (459 ± 23 ms), and D5 (455 ms ± 26, all *p*s < 0.01, Newman-Keuls corrected)—when sounds were perceived close to the body. The spatial modulation of tactile perception due to sound position indicates that the critical spatial range where sounds became effective in modulating tactile RTs were localized between D2 and D3, suggesting that, normally, the boundaries of PPS representation around the upper limb could be localized at that location (also see Canzoneri et al., 2012, 2013a,b). Interestingly, those boundaries were extended after the Synchronous training, as shown by a change in the function describing the relationship between the perceived sound position and tactile RTs. After the training, indeed, RTs at D2 (470 ms ± 23), associated with a previously perceived far position in space, were no more significantly different as compared to RTs in D3 (450 ms ± 22), D4 (439 ms ± 22), and D5 (439 ms ± 22), but they were statistically different only from RTs at D1 (*p* < 0.03). Thus, the critical spatial range where sounds became effective in modulating tactile RTs shifted after the training up to include positions more distant from the hand, i.e., between D2 and D1, whereas it was located between D3 and D2 before the training. Indeed, RTs at D2, and not at any other distance, were significantly faster after synchronous training as compared to before training (*p* < 0.02).

For the Asynchronous training condition, the ANOVA conducted on RTs with Session (Before Training, After Training), Sound (IN, OUT) and Distance (from D1 to D5) showed a significant Sound × Distance interaction [*F*_(4, 60)_ = 6.86, *p* < 0.001; η^2^ = 0.31]. The pattern of results both for IN and OUT sounds mirrors the same effect found for the Synchronous condition before training: as sound distance from the body decreased, RTs progressively shortened. Newman-Keuls *post-hoc* comparisons confirmed this effect: for the IN sound tactile RTs at D1 (Mean RTs ± S.E.M, 484 ms ± 24) and D2 (481 ms ± 26), when the sound was perceived far from the body, were slower compared to RTs at D3 (444 ms ± 25), D4 (440 ms ± 28), and D5 (433 ms, ± 25, all *p*s <0.01), when the sound was perceived close to the body (data for the OUT sound are reported in Table 1). Importantly, the space dependent modulation of RTs due to sound position was not different before and after the training session, as the three-way interaction Session × Sound × Distance was not significant [*F*_(4, 60)_ = 0.39, *p* = 0.82, η^2^ = 0.02], as well as the main effect of Session [*F*_(1, 15)_ = 1.98, *p* = 0.18, η^2^ = 0.12]. Thus, the boundaries of PPS representation were localized between D2 and D3, both before and after the training (see Figure 7B).

## Discussion

Since Iriki's (1996) seminal paper, neurophysiological, neuropsychological and behavioral studies have supported the view that the experience of using a tool to act upon a portion of space, normally not reachable by the upper limb, extends PPS representation (see for reviews, Maravita and Iriki, 2004; Làdavas and Serino, 2008). Although this conclusion is generally accepted (but see Holmes, 2012 and below for an alternative view), the mechanism underlying PPS extension due to tool-use is largely unknown.

Here we test the prediction, generated by our neurocomputational approach, that extension of PPS is the consequence of a mechanism capturing the synchronicity between a tactile stimulus at the hand and an auditory (or visual) stimulus in the far space, even without any use of the tool. In term of sensory inputs to the brain, this synchronous stimulation mimics the sensory-motor consequences of tool-use: while using the tool, an auditory (and/or visual) stimulus from the space, where the tool is operated, is temporally correlated with a tactile stimulation at the hand, transmitted via the tool. When the neural network is fed with tactile stimulation at the hand and synchronous auditory stimulation from the far space, the auditory RF of multisensory neurons extends toward the far space, producing an extension of PPS representation. In contrast, audio-tactile stimulation with a reduced temporal correlation between the two stimuli, i.e., asynchronous stimulation, is ineffective. Results from the simulation experiment presented here (Figures 4, 5) clearly support this view, showing that after a synchronous, but not an asynchronous audio-tactile training, the multisensory area of the neural network was activated by auditory stimuli presented at farther locations of space, which therefore boosted responses to tactile stimuli, as compared to before the training.

Results from the *in vivo* behavioral study (Figure 7) confirmed the prediction generated and tested by means of our neural network model. In a group of healthy participants, we simulated the sensory consequence of a tool-use training as predicted by the model by providing synchronous tactile stimulation at the hand and auditory stimulation from the far space, as it aroused from the tip of a tool, although participants were not using nor even holding a tool. We measured the extension of PPS representation around the hand before and after such training, as well as before and after a control training with asynchronous stimulation. In line with previous evidence (Canzoneri et al., 2012, 2013a,b; Teneggi et al., 2013; Noel et al., 2014), we showed that a sound approaching the body speeds up RT for tactile stimuli as far the sound is perceived at a given distance from the body. Such distance can be considered the boundary of PPS representation. The PPS boundary was localized at a farther position of space after the synchronous training as compared to before the training. On the contrary, after the asynchronous training, the PPS boundaries did not move to farther distance, thus showing that the temporal coincidence between the tactile stimulus at the hand and the auditory stimulus from the far space during the training is a necessary condition for extending PPS representation.

The present behavioral results and the computational findings obtained here and in our previous studies (Magosso et al., 2010b) are compatible with neurophysiological results in monkeys showing that hand-centered visual RFs of neurons located in the intraparietal sulcus elongated after that monkeys used a rake to retrieve pieces of food placed in the far space (Iriki et al., 1996). This effect has been attributed to the formation of new functional synapses from high-order visual areas to the intraparietal cortex (Ishibashi et al., 2002; Hihara et al., 2006). Such neurophysiological effect can be considered the biological counter-part of the strengthening of synapses from unimodal visual or auditory areas to multisensory areas based on a Hebbian-like mechanism predicted by our neural network model, and supported by the present experimental findings.

The striking result from the present study is that a change in PPS representation, mimicking that obtained after tool-use, can be evoked also when a tool is neither actually used nor present. A key factor, instead, is feeding the neural network with the same sensory stimulation produced by tool-use activity, in the present case the auditory stimulation due to the sound produced by the tool when hitting an object placed in the far space and the concurrent tactile stimulation at the hand handling the tool. This conclusion might be surprising with respect to the dominant view in the literature suggesting that *actively using* the tool is necessary for extending PPS representation, since a prolonged, but passive exposure to a tool have no effect (Farnè and Làdavas, 2000; Maravita et al., 2001). The latter view fits with a standard definition of tool as an object physically and actively used to act upon other objects (see Beck, 1980; Holmes and Spence, 2006). However, previous and the present findings show that PPS representation can extend also in conditions of virtual, not physical interaction, and even in absence of action. For instance, Bassolino et al. (2010) demonstrated that using a mouse, a technological device which establishes a virtual—but not physical—connection between near and far space (Goldenberg and Iriki, 2007), extended PPS representation from the space around the hand to that around the computer screen where the mouse cursor was operated. In that case, however, a tool was still present and participants had active experience of tool-use. In the present study, instead, PPS extension was obtained in absence of action. Taken together these findings suggest that neither a physical, nor a functional inter-action between near and far space is necessary to extend PPS representation. Rather, the sensory-feedback linked to tool-use seems a sufficient factor to trigger plasticity in PPS representation: thanks to a Hebbian-like mechanism, after prolonged synchronous tactile stimulation at the hand and multisensory stimulation from the far space, multisensory areas associate the two stimuli, as if they occurred from a functionally equivalent sector of space. Note, however, that we are not claiming that action is irrelevant for PPS extension after tool-use. We are rather suggesting that, under natural conditions, PPS areas process the multisensory consequences of actions related to tool-use, i.e., contingent and temporally correlated near and far stimulation, and that those inputs are critical for plasticity in PPS representations.

It is also important to acknowledge that other authors question the idea that the effects of tool-use depend on a change in the receptive field of PPS neurons, by proposing instead, that the tool-use results in an automatic, multisensory, shift of spatial attention to the space where the tool exerts its effect (see e.g., Holmes et al., 2007; Holmes, 2012). Our neural network model can account for attentional effects of tool-use. Prolonged tool use, or related stimulation, might increase crossmodal attention at far portions of space because after tool-use multisensory neurons are more likely to be activated by far stimuli, in any modality. On the other hand, however, an explanation based on attentional shift cannot explain why such effect arises after synchronous, but not after asynchronous near–far stimulation. In fact, a pure attentional account should generate the opposite prediction, because in condition of synchronous near and far stimulation, two different sectors of space are concurrently activated, and spatial attention is therefore deployed at the two locations, rather than shifting toward the far space. For these reasons, we believe that the interpretation of the effects of tool-use proposed in the present paper is more parsimonious, more able to account for multiple results and more plausible from a neurophysiological point of view than an explanation only based on the shift of spatial attention.

This kind of stimulation embedded in our training is reminiscent of the paradigms used to induce the Rubber Hand Illusion (RHI). In RHI experiments, a realistic fake hand can be perceived as a part of one's own body if concurrent visuo-tactile stimulation is seen on the fake hand and felt on one's own hand, hidden from view (Botvinick and Cohen, 1998). Ehrsson and colleagues (Ehrsson et al., 2005; Makin et al., 2008; Ehrsson, 2012) proposed that the illusory body parts ownership that people experience during the RHI might involve bimodal and/or trimodal neurons in premotor and parietal cortices that normally respond only to stimuli presented within one's own PPS. Recently, Blanke (2012) suggested that, during the RHI, seeing the rubber hand being stimulated and experiencing a synchronous tactile stimulation on one's own hand triggers a shift of the receptive field of bimodal neurons toward the fake body part. This effect might generate a change in body perception, that is the rubber hand is perceived as the real hand. This interpretation of the RHI is in line with a recent fMRI study of Brozzoli et al. (2012) measuring how activity of human premotor and parietal cortices coding the PPS varies in response to visual stimuli presented close to the subject's real hand or to a rubber hand, placed at a distance. They found that only when subjects experienced ownership for the rubber hand, through induction of the RHI, PPS areas responded also to stimuli presented close to the rubber hand. These findings suggest that visual RF of multisensory neurons extended to incorporate the rubber hand into PPS representation. Results from the present study offers a simple explanation for Brozzoli et al.'s findings and might inspire neural models explaining the RHI: synchronicity between tactile stimulation at the hand and auditory or visual stimulation from a space location other than that of the hand seems a sufficient condition to trigger PPS extension. This mechanism, however, does not necessarily imply any sense of ownership for stimuli at the far space, being those stimuli a rubber hand, a tip of a tool, or even an empty space.

To sum up, the present study offers empirical support to the unconventional hypothesis, generated by a neural network model, that plasticity in PPS representation after tool-use does not strictly depend on the function of the tool nor from the actions performed with the tool, but it is triggered by the sensory feedback of tool-use, i.e., synchronous tactile stimulation at the hand, due to holding the tool, and multisensory (auditory or visual) stimulation from the far space, where the tool is operated.

### Conflict of interest statement

The authors declare that the research was conducted in the absence of any commercial or financial relationships that could be construed as a potential conflict of interest.
