# Supplementary material for: Extending peripersonal space representation without tool-use: evidence from a combined behavioral-computational approach
Source: Front Behav Neurosci. 2015 Feb 2;9:4. doi: 10.3389/fnbeh.2015.00004 (PMC4313698; doi:10.3389/fnbeh.2015.00004)
Supplement: Supplementary file 1 [file Table1.DOCX]

Serino et al., Extending peripersonal space representation without tool-use: evidence from a combined behavioural-computational approach

**SUPPLEMENTARY MATERIAL**

***1. Quantitative model description***

Conceptually the model is similar to our previous one concerning visuo-tactile interaction (Magosso et al. Neuropsychologia 2010). Some modifications have been included in order to deal with audio-tactile interaction rather than visuo-tactile one. In this section, model equations, parameter values and simulation details are reported.

In the following, the superscript *t*, *a* and *m* will indicate quantities referring to tactile, auditory and multisensory neurons, respectively. The subscripts *ij* or *hk* will represent the spatial position of individual neurons.

The unisensory neurons

The tactile unisensory area is composed by *Mt* = 40 x *Nt* = 20 neurons; the auditory area is composed by *Ma* = 20 x *Na* = 3 neurons. RFs of tactile neurons are arranged at a distance of 0.5 cm along both dimensions (so they code for a portion of skin of 20 cm x 10 cm); RFs of auditory neurons are arranged at a distance of 10 cm (so they cover a portion of auditory space of 200 cm x 30 cm on and around the hand). In the following, *xj* and *yj* will indicate the centre of the RF of a generic neuron *ij*. By considering a reference frame rigidly connected with the hand (see Fig. 1), we have

$x_{i}^{t}=i\cdot0.5$ cm (i = 1,2,…,*Mt*) $y_{j}^{t}=j\cdot0.5$ cm (j = 1,2,…,*Nt*)

for the tactile neurons, and

$x_{i}^{a}=i\cdot10$ - 5 cm (i = 1,2,…,*Ma*) $y_{j}^{a}=j\cdot10$ cm – 15 cm (j = 1,2,…,*Na*)

for the auditory neurons.

A generic neuron within an unisensory area receives three different inputs:

i) The input (say $\phi_{ij}(t)$) due to the external stimulus. $\phi_{ij}(t)$ is computed as the inner product between the external stimulus and the neuron receptive field:

$\phi_{ij}^{s}(t)=\int_{\Omega_{x}} \int_{\Omega_{y}} \Phi_{ij}^{s}(x,y)\cdot I^{s}(x,y,t)dxdy\cong\sum_{l} \sum_{n} \Phi_{ij}^{s}(x_{l},y_{n})\cdot I^{s}(x_{l},y_{n},t)\Delta x_{l}\Delta y_{n}$ (1)

*s = t, a*

where $I^{s}(x,y,t)$ is the external stimulus (tactile or auditory) applied at the coordinates *x*, *y* and at time *t*, and *Ω_x_* and *Ω_y_* are the *x* and *y* domains. The right hand member of Eq. (1) means that the integral is computed with the histogram rule by discretizing the *x* and *y* domain (with *Δxl = Δyn* = 0.2 cm).

The receptive field of unisensory neurons (say $\Phi_{_{ij}}^{s}(x,y)$) is described via a Gaussian function, i.e.

$\Phi_{ij}^{s}(x,y)=\Phi_{0}^{s}\cdot\exp\left(-\frac{(x_{i}^{s}-x)^{2}+(y_{j}^{s}-y)^{2}}{2\cdot(\sigma_{\Phi}^{s})^{2}}\right)$, *s* = *t*, *a* (2)

where *xi*, *yj* is the centre of the RF, *x* and *y* are generic spatial coordinates, $\Phi_{0}^{s}$ and $\sigma_{\Phi}^{s}$ represent the amplitude and standard deviation of the Gaussian function. To account for the low spatial resolution of the auditory system, RFs of auditory neurons are assumed large, in particular $\sigma_{\Phi}^{a}$ >> $\sigma_{\Phi}^{t}$.

Both the auditory and visual inputs are mimicked via a l Gaussian function:

$I^{s}(x,y,t)=\left\{\begin{matrix} 0,\text{ t}<t_{0} \\ (I_{0}^{s}+\sigma_{\nu}^{s}\cdot\nu)\cdot\exp\left(-\frac{(x_{0}^{s}-x)^{2}+(y_{0}^{s}-y)^{2}}{2\cdot(\sigma_{I}^{s})}\right),\text{ t}\geq\text{ t}{}_{0} \end{matrix}\right.$ *s = t, a* (3)

*t_0_* is the instant of stimulus application, *x_0_, y_0_* is the central point of the stimulus. The amplitude of the Gaussian function ($I_{0}^{s}+\sigma_{\nu}^{s}\cdot\nu$) mimics the stimulus intensity: it is affected by a Gaussian random noise $\sigma_{\nu}^{s}\cdot\nu$ with standard deviation $\sigma_{\nu}^{s}$ and zero mean ($\nu$ is the standard normal variable). $\sigma_{I}^{s}$ sets the spatial extension of the stimulus. $\sigma_{I}^{s}$ (s = t, a) was assigned a small value (= 0.3 cm) to simulate localized tactile or auditory inputs. The random noise in stimulus intensity was included to give rise to network variability in response to the same stimulation.

ii) The lateral input (say $l_{ij}(t)$) that the unisensory neuron receives from other neurons in the same area via lateral synapses. This is computed as:

$l_{ij}^{s}(t)=\sum_{h=1}^{N^{s}} \sum_{k=1}^{M^{s}} L_{ij,hk}^{s}\cdot z_{hk}^{s}(t)$ *s* = *t,* *a* (4)

$z_{hk}^{s}(t)$ represents the activity of the *hk* neuron in the area *s* (*s = t, a*) computed below. $L_{ij,hk}^{s}$ indicates the strength of the synaptic connection from the pre-synaptic neuron at the position *hk* to the post-synaptic neuron at the position *ij*. These synapses are symmetrical and are arranged according to a “Mexican hat” function (obtained as the difference between two Gaussian functions):

$L_{ij,hk}^{s}=\left\{\begin{matrix} \mathbf{L}_{\mathbf{ex}}^{\mathbf{s}}\cdot\mathbf{exp}\left(-\frac{\left(\mathbf{D}_{\mathbf{x}}^{\mathbf{s}}\right)^{\mathbf{2}}+\left(\mathbf{D}_{\mathbf{y}}^{\mathbf{s}}\right)^{\mathbf{2}}}{\mathbf{2}\cdot\left(\mathbf{\sigma}_{\mathbf{ex}}^{\mathbf{s}}\right)^{\mathbf{2}}}\right)-\mathbf{L}_{\mathbf{in}}^{\mathbf{s}}\cdot\mathbf{exp}\left(-\frac{\left(\mathbf{D}_{\mathbf{x}}^{\mathbf{s}}\right)^{\mathbf{2}}+\left(\mathbf{D}_{\mathbf{y}}^{\mathbf{s}}\right)^{\mathbf{2}}}{\mathbf{2}\cdot\left(\mathbf{\sigma}_{\mathbf{in}}^{\mathbf{s}}\right)^{\mathbf{2}}}\right)\mathbf{ ij}\neq\mathbf{hk} \\ \mathbf{0 ij}=\mathbf{hk} \end{matrix}\right.$ (5)

*s* = *t,* *a*

Parameters $D_{x}^{s}$ and $D_{y}^{s}$represent the distances between the pre-synaptic and post-synaptic neurons along the horizontal and vertical coordinates. Parameters $L_{ex}^{s}$ and $\sigma_{ex}^{s}$ define the excitatory Gaussian function, parameters $L_{in}^{s}$ and $\sigma_{in}^{s}$ the inhibitory one: they establish the strength and extension of these synapses. To have a Mexican hat disposition, the following conditions were satisfied: $L_{ex}^{s}$>$L_{in}^{s}$ and $\sigma_{ex}^{s}$<$\sigma_{in}^{s}$. The null term in Eq. 5 avoids auto-excitation. To prevent undesired border effects, synapses were realized by a circular structure so that every neuron of each area receives the same number of side connections. Lateral synapses parameters were given the same values in the two unisensory area: they were assigned to reach a trade-off between excitation and inhibition, so that an external stimulus produces the activation of a limited number of unisensory neurons, avoiding that excitation propagates in an uncontrolled manner to the overall area.

iii) The feedback input (say $b_{ij}(t)$) from the multisensory neuron via the feedback synapses. This term has the following expression:

$b_{ij}^{s}(t)=B_{ij}^{s}\cdot z^{m}(t)$, *s = t, a* (6)

$z^{m}(t)$ represents the activity of the multisensory neuron (computed below). $B_{ij}^{s}$ indicates the strength of the synaptic connection from the pre-synaptic multisensory neuron to the post-synaptic unisensory neuron at the position *ij* in area *s* (*t, a*).

We supposed that the feedback synapses reach all tactile neurons and have the same value independently of the position of the tactile neuron in the area:

$B_{ij}^{t}=B_{0}^{t},\text{ } \forall i =1,2,...,M^{t}\text{ }\forall j =1,2,...,N^{t}$ (7)

Conversely, the synapses (both feedback and feedforward) connecting the auditory unisensory neurons and the multisensory neuron depend on the position of the auditory neuron’s RF with respect to the hand. In particular, these synapses assume a constant value for the auditory neurons coding for the space on and near the hand (within a distance of 40 cm from the hand); then their value decreases exponentially. Accordingly, we have:

$B_{ij}^{a}=\alpha\cdot B_{0}^{a}\cdot\exp\left(-\frac{D_{ij}}{k_{1}}\right)+(1-\alpha)\cdot B_{0}^{a}\cdot\exp\left(-\frac{D_{ij}}{k_{2}}\right)$, (8)

Dij is equal to zero for the auditory neurons having RF’s centre $x_{i}^{a}\in\left\{0\div60\text{ cm}\right\}$, $y_{j}^{a}\in\left\{-10\div20\text{ cm}\right\}$ whereas, for auditory neurons having RFs outside this region, Dij is computed as the minimum Euclidean distance of the RF centre from the boundaries of this region.

$B_{0}^{a}$ denotes the value of the feedback synapses when *Dij* = 0 (i.e., it is the strength of the feedback synapses targeting auditory neurons coding the space *on and near* the hand). $B_{0}^{a}$ and $B_{0}^{t}$ were given the same value: this was set sufficiently small to avoid that the activation of the multisensory neuron due to an unisensory external stimulus produces a phantom activation in the other un-stimulated area, and sufficiently high so that - in case of multisensory stimulation - a stimulus in one modality may reinforce activation in the other unisensory area. Parameters *k_1_* and *k_2_* are the fast and slow decay rate, respectively and, *α* is a parameter setting the relative amplitude of each exponential. *k_1_, k_2_* and *α* were assigned so that synaptic strength decreases rapidly immediately outside of the near-hand region, and then exhibits a slower decrease.

The activity ($z_{^{ij}}(t)$) of a unisensory neuron is computed from its overall input through a first order equation and a sigmoidal relationship:

$\tau\frac{dq_{ij}^{s}(t)}{dt}=-q_{ij}^{s}(t)+u_{ij}^{s}(t)$ *s = t, a* (9)

$z_{ij}^{s,L}(t)=\Psi(q_{ij}^{s}(t))\cdot H\left(\Psi(q_{ij}^{s}(t))\right)$ *s = t, a* (10)

$\Psi\left(q_{ij}^{s}(t)\right)=\frac{f_{\min}^{s}+f_{\max}^{s}\cdot e^{\left(q_{ij}^{s}-q_{c}^{s}\right)\cdot r^{s}}}{1+e^{\left(q_{ij}^{s}-q_{c}^{s}\right)\cdot r^{s}}}$ (11)

$u_{^{ij}}^{s}(t)$ is the overall neuron input (computed as the sum of the three contributions $\phi_{^{ij}}^{s}(t)$, $l_{^{ij}}^{s}(t)$, $b_{^{ij}}^{s}(t)$). $q_{^{ij}}^{s}(t)$ is the state variable, and *Ψ(·)* is a sigmoidal function. *H(·)* is the Heaviside function introduced to avoid that neuron activity becomes negative. *τ* is the time constant of the differential equation; its value was set within the range of membrane time constants reported in the literature [Dayan, P., & Abbott, L. F. (2001). Theoretical neuroscience (Vol. 31). Cambridge, MA: MIT press]. *fmin* and *fmax* are the lower and upper saturation values of the sigmoidal relationship, $q_{c}^{s}$ denotes the central point of the sigmoid, and *rs* sets the central slope of the sigmoid. The upper saturation value *fmax* is set to 1 (i.e., neuron activity is normalized to the maximum). The other parameters of the sigmoid were assigned so that neuron activity has an elevated threshold (below which neuron remains silent) and a smooth transition from silence to saturation, to help controlling excitation in the unisensory areas.

The multisensory neuron

The multisensory neuron receives inputs from the neurons in the unisensory areas via feedforward synapses, according to the following equation:

$u^{m}(t)=\sum_{i=1}^{N^{t}} \sum_{j=1}^{M^{t}} W_{ij}^{t}\cdot z_{ij}^{t}(t)+\sum_{i=1}^{N^{a}} \sum_{j=1}^{M^{a}} W_{ij}^{a}\cdot z_{ij}^{a}(t)$ (12)

$z_{ij}^{s}(t)$ (*s = t, a*) represents the activity of the neuron *ij* in the unisensory (tactile or auditory) area, computed through Eq. 9-11. $W_{ij}^{s}$ (*s = t, a*) denotes the feedforward synapses from the unisensory neuron *ij* to the multisensory neuron. The feedforward synapses have the same arrangement as the feedback one:

$W_{ij}^{t}=W_{0}^{t},\text{ } \forall i =1,2,...,N^{t}\text{ }\forall j =1,2,...,M^{t}$ (13)

$W_{ij}^{a}=\alpha\cdot W_{0}^{a}\cdot\exp\left(-\frac{D_{ij}}{k_{1}}\right)+(1-\alpha)\cdot W_{0}^{a}\cdot\exp\left(-\frac{D_{ij}}{k_{2}}\right)$ (14)

where the meaning of the symbols is the same as in Eq. 7 and 8.

The activity of the multisensory neuron is obtained from its input by using equations similar to Eqs. 9 and 11:

$\tau\frac{dq^{m}(t)}{dt}=-q^{m}(t)+u^{m}(t)$ (15)

$z^{m}(t)=\Psi(q^{m}(t))\cdot H\left(\Psi(q^{m}(t))\right)$ (16)

$\Psi\left(q^{m}(t)\right)=\frac{f_{\min}^{m}+f_{\max}^{m}\cdot e^{\left(q{}^{m,L}-q_{c}^{m}\right)\cdot r^{m}}}{1+e^{\left(q{}^{m,L}-q_{c}^{m}\right)\cdot r^{m}}}$ (17)

The differential equations are numerically solved using the Euler integration method with a discrete time step *Δt* = 0.4 ms.

***2 Model training***

During the training, a tactile stimulus and an auditory stimulus are applied to the network. The tactile stimulus is described by Eq. 3 and is applied at the centre of the hand ($x_{0}^{t}=10$ cm, $y_{0}^{t}=5$ cm, although the position of the tactile stimulus is irrelevant). Parameters of the stimulus intensity ($I_{0}^{t}+\sigma_{\nu}^{t}\cdot\nu=2.5+0.1\cdot\nu$) were set so that the tactile stimulus by itself is able to activate both the tactile area and the multisensory neuron about the upper saturation level, with a gradual activation of the network up to the final new steady-state.

The auditory stimulus is described by Eq. 3 and is applied far from the hand, at position $x_{0}^{a}=100$ cm, $y_{0}^{a}=5$ cm, so that it is not able to activate by itself the multisensory neuron. Parameters of the stimulus intensity ($I_{0}^{a}+\sigma_{\nu}^{a}\cdot\nu=3.6+0.4\cdot\nu$) were assigned so that the stimulus produces a strong activation of auditory neurons.

Each stimulus lasted 1000 simulation steps (i.e., 400 ms).

To train the network, the audio-tactile stimulation was presented 30 times to the network. In case of the tool-use training the tactile and the auditory stimuli were always applied simultaneously (i.e., stimulus onset asynchrony, SOA = 0 ms), i.e, we called this *synchronous training*. In case of the *asynchronous training*, the tactile and auditory stimuli were applied with a SOA randomly varying between 200 ms and 600 ms (so that the two stimuli were partially superimposed or completed separated in time). During each presentation of the audio-tactile stimulation (synchronous or asynchronous), the feedforward synapses from a generic unisensory neuron *ij* (in the tactile or auditory area) were trained according to the following Hebbian rule:

$W_{ij}^{s}(t+T)=W_{ij}^{s}(t)+\Delta W_{ij}^{s}(t)$ *s = t, a* (18)

$\Delta W_{ij}^{s}(t)=\rho_{ij}^{s}\left(t\right)\cdot z_{ij}^{s}(t)\cdot(z^{m}(t)-\theta)^{+}-k^{s}\cdot(z^{m}(t)-\theta)^{+}\cdot\left(W_{ij}^{s}(t)-W_{ij}^{s}(0)\right)$ *s = t, a* (19)

where $(\cdot)^{+}$ indicate the positive part function.

*T* in Eq. 18 is the temporal step of synapses updating. The Hebbian rule contains a reinforcing factor (first term in the right-hand member of Eq. 19) and a forgetting factor (second term in the right-hand member of Eq. 19). According to the reinforcing factor, the synapsis increases when both the pre-synaptic (unisensory) neuron and the post-synaptic (multisensory) neuron are active. In particular, the post-synaptic multisensory activity is compared with a small threshold (5% of the maximum activation), in order to avoid a reinforcement in case of very small activity of the post-synaptic neuron. $\rho_{ij}^{s}(t)$ denotes the reinforcement learning factor (see below). The forgetting factor acts only on the newly created synapses: synapsis forgets parts of its value when only the post-synaptic neuron is active (above threshold) without a simultaneous activation of the pre-synaptic neuron. $W_{ij}^{s}(0)$ indicates the pre-existing value of the synapsis (i.e. synapsis value in basal condition). *k* sets the forgetting rate. To avoid that synapsis increases unlimitedly, we imposed a saturation constraint for synapsis value; the reinforcement learning factor ($\rho_{ij}^{s}(t)$) progressively reduces to zero as synapses approach their maximum value. We have:

$$\rho_{ij}^{s}(t)=\rho_{0}^{s}\cdot\left(W_{\max}^{s}-W_{ij}^{s}(t)\right)$$

$W_{\max}^{s}$ *(s = t, a)* is the maximum value allowed for the tactile and auditory synapses, and is assumed equal to the pre-existing value of the synapses on the hand, that is $W_{\max}^{t}=W_{0}^{t}$ and $W_{\max}^{a}=W_{0}^{a}$. Therefore, tactile feedforward synapses (as well as auditory feedforward synapses on and close the hand) are not subjected to modifications.

Value of $\rho_{0}^{s}$and $k^{s}$were assigned so that to have a gradual modification of the synapses.

***3 Assessment of training effects on PPS representation in the model***

Extension of PPS representation was evaluated before training (i.e., synapses at their pre-existing basal value), at the end of the tool-use training (audio-tactile synchronous training) and at the end of the asynchronous training. Extension of PPS was evaluated in two conditions:

i) Unisensory conditions, by assessing the activation of the multisensory neuron (see Fig. 4A and 5A). An unisensory auditory stimulus was applied at different distances from the hand. The external stimulus was mimicked according to Eq. (3) with a small standard deviation ($\sigma_{I}^{s}$ =0.3 cm) to replicate a localized stimulus in the space; the y coordinate of the stimulus position ($y_{0}^{t}$) was always 5 cm; whereas the x coordinate of the stimulus position ($x_{0}^{t}$) was varied between 160 cm (140 cm distance from the hand) to 20 cm (0 cm distance from the hand) in 10 cm steps. The stimulus lasted 400 ms. For each position, the stimulus, having intensity affected by random noise ($3.6+0.4\cdot\nu$), was presented 30 times to the network, and the final activation of the multisensory neuron was considered for any single simulation. For each stimulus position, the mean value of the multisensory neuron’s activations and the standard error of the mean were computed and displayed.

ii) Multisensory conditions, by computing the network tactile RT (see Fig. 4B and Fig. 5B). A tactile stimulus, having intensity affected by random noise ($2.5+0.1\cdot\nu$), was applied on the hand ($x_{0}^{t}=10$ cm, $y_{0}^{t}=5$ cm) together with a simultaneous auditory stimulus. The auditory stimulus had intensity ($3.6+0.4\cdot\nu$ ), and was placed at different distances from the hand as in unisensory conditions (see point i) above). Both the auditory and tactile stimuli lasted 400 ms. For each position, the audio-tactile stimulation was presented 30 times to the network. For each simulation, we computed the network tactile RT as the time necessary for the overall tactile area to reach the 90% of its final (steady-state) activation. The following computation was applied:

$Z^{t}(t)=\sum_{i=1}^{M^{t}} \sum_{j=1}^{N^{t}} z_{ij}^{t}(t)\text{ }\forall\text{ t }\in\text{ }\left\{\text{0 }\div\text{ 400 ms}\right\}$ (20)

$RT\text{ }\in\text{ }\left\{\text{0 }\div\text{ 400 ms}\right\}\text{ : }Z^{t}(RT)=0.9\cdot\text{ }Z^{t}(t=400\text{ ms})\text{ }$ (21)

Eq. 20 calculates the overall activity in the tactile area at each simulation time. Network tactile RT (Eq. 21) is computed as the simulation time at which the overall tactile activation reached the 90% of its final (steady-state) activation $Z^{t}(t=400\text{ ms})\text{ }$.

The values of the network RT obtained at the different sound distances (30 x 15 = 450 points), before training and after training, were fitted – in the least square sense - with a sigmoidal function having the following equation

$f_{RT}(D)=\frac{\eta_{\min}+\eta_{\max}\cdot e^{\left(\frac{D-D_{c}}{h}\right)}}{1+e^{\left(\frac{D-D_{c}}{h}\right)}}$ (22)

*D* is the distance from the hand and represents the independent variable of the function; $\eta_{\min}$ and $\eta_{\max}$ are the lower and upper saturation of the sigmoidal function; *Dc* is the central point of the sigmoid, i.e. the point at which the sigmoid assumes value $\left(\eta_{\min}+\eta_{\max}\right)/2$, and *h* sets the slope of the sigmoid at the central point.

All the four parameters of the function ($\eta_{\min}$,$\eta_{\max}$, *Dc*, *h*) were estimated by the fitting procedure, and confidence intervals were computed too. The estimated value of the sigmoid central point (*Dc*) was assumed as the boundary of the PPS.

*Table 1 – Values of model parameters*

*Unisensory neurons receptive fields*

$\Phi_{0}^{t}$ = 1 $\sigma_{\Phi}^{t}$ = 0.5 cm $\Phi_{0}^{a}$ = 1 $\sigma_{\Phi}^{a}$ = 10 cm

*External stimuli*

$I_{0}^{t}$ = 2.5 $\sigma_{\nu}^{t}$ = 0.1 $I_{0}^{a}$ = 3.6 $\sigma_{\nu}^{a}$ = 0.4

$\sigma_{I}^{t}$ = 0.3 cm $\sigma_{I}^{a}=0.3$cm

*Lateral synapses in unisensory areas*

$L_{ex}^{t}$ = 0.15 $L_{in}^{t}$ = 0.05 $\sigma_{ex}^{t}$ = 1 cm $\sigma_{in}^{t}$ = 4 cm

$L_{ex}^{a}$ = 0.15 $L_{in}^{a}$ = 0.05 $\sigma_{ex}^{a}$ = 20 cm $\sigma_{in}^{a}$ = 80 cm

*Feedforward and feedback synapses*

$W_{0}^{t}$ = 6.5 $W_{0}^{a}$ = 6.5 $B_{0}^{t}$ = 2.5 $B_{0}^{a}$ = 2.5

*k_1_*= 10 cm *k_2_* = 700 cm *α* = 0.9

*Input-output relationship of unisensory neurons*

$f_{\min}^{t}$ = -0.12 $f_{\max}^{t}$ = 1 $q_{c}^{t}$= 19.43 $r^{t}$= 0.34

$f_{\min}^{a}$ = -0.12 $f_{\max}^{a}$ = 1 $q_{c}^{a}$= 19.43 $r^{a}$= 0.34

$\tau$ = 20 ms

*Input-output relationship of the multisensory neurons*

$f_{\min}^{m}$ = 0 $f_{\max}^{m}$ = 1 $q_{^{c}}^{m}$= 12 $r^{m}$= 0.6

$\tau$ = 20 ms

*Hebbian rule*

$W_{\max}^{t}$ = 6.5 $W_{\max}^{a}$ = 6.5 $\rho_{0}^{t}$ = 3.85·10^-4^ $\rho_{0}^{a}$ =3.85·10^-4^

$k^{t}$= 5·10^-5^ $k^{a}$= 5·10^-5^ *θ = 0.05*
